# Analgesic tolerance of opioid agonists in mutant mu-opioid receptors expressed in sensory neurons following intrathecal plasmid gene delivery

**DOI:** 10.1186/1744-8069-9-63

**Published:** 2013-12-05

**Authors:** Guangwen Li, Fei Ma, Yanping Gu, Li-Yen Mae Huang

**Affiliations:** 1 Department of Neuroscience and Cell Biology, University of Texas Medical Branch, 301 University Boulevard, Galveston, TX 77555-1069, USA; 2 Department of Physiology, University of Kentucky, 800 Rose Street, Lexington, KY 40536-0298, USA

**Keywords:** Opioid tolerance, Opioid receptors, T394A mutation, Dorsal root ganglion, Nociception, Plasmid DNA injection

## Abstract

**Background:**

Phosphorylation sites in the C-terminus of mu-opioid receptors (MORs) are known to play critical roles in the receptor functions. Our understanding of their participation in opioid analgesia is mostly based on studies of opioid effects on mutant receptors expressed in *in vitro* preparations, including cell lines, isolated neurons and brain slices. The behavioral consequences of the mutation have not been fully explored due to the complexity in studies of mutant receptors *in vivo.* To facilitate the determination of the contribution of phosphorylation sites in MOR to opioid-induced analgesic behaviors, we expressed mutant and wild-type human MORs (hMORs) in sensory dorsal root ganglion (DRG) neurons, a major site for nociceptive (pain) signaling and determined morphine- and the full MOR agonist, DAMGO,-induced effects on heat-induced hyperalgesic behaviors and potassium current (I_K_) desensitization in these rats.

**Findings:**

A mutant hMOR DNA with the putative phosphorylation threonine site at position 394 replaced by an alanine (T394A), i.e., hMOR-T, or a plasmid containing wild type hMOR (as a positive control) was intrathecally delivered. The plasmid containing GFP or saline was used as the negative control. To limit the expression of exogenous DNA to neurons of DRGs, a neuron-specific promoter was included in the plasmid. Following a plasmid injection, hMOR-T or hMOR receptors were expressed in small and medium DRG neurons. Compared with saline or GFP rats, the analgesic potency of morphine was increased to a similar extent in hMOR-T and hMOR rats. Morphine induced minimum I_K_ desensitization in both rat groups. In contrast, DAMGO increased analgesic potency and elicited I_K_ desensitization to a significantly less extent in hMOR-T than in hMOR rats. The development and extent of acute and chronic tolerance induced by repeated morphine or DAMGO applications were not altered by the T394A mutation.

**Conclusions:**

These results indicate that phosphorylation of T394 plays a critical role in determining the potency of DAMGO-induced analgesia and I_K_ desensitization, but has limited effect on morphine-induced responses. On the other hand, the mutation contributes minimally to both DAMGO- and morphine-induced behavioral tolerance. Furthermore, the study shows that plasmid gene delivery of mutant receptors to DRG neurons is a useful strategy to explore nociceptive behavioral consequences of the mutation.

## Background

Morphine is one of the most frequently used analgesic opioids used in clinics for the treatment of severe acute and chronic pain. Its use is often limited by a reduced effectiveness to elicit pain relief following repeated use i.e., tolerance. As the result of the involvement of complex molecular events, the mechanisms underlying morphine tolerance *in vivo* remain unclear. Nearly all the opioids used in clinics, including morphine and fentanyl, exert their analgesic effects through the activation of mu-opioid receptors (MORs). Once activated by opioids, the MOR undergoes G-protein receptor kinase (GRK)-dependent phosphorylation. Within minutes of agonist exposure, MOR starts to lose coupling with its effectors, including adenylyl cyclase, G protein-coupled inwardly rectifying potassium (GIRK) channels and voltage dependent Ca^2+^ channels, and reduces its agonist sensitivity, a phenomenon referred to as “desensitization” [1,2]. Following the removal of agonists, MOR quickly resensitizes and dephosphorylates. After a long (hrs to weeks) opioid exposure, however, MOR desensitization becomes enhanced and receptor-mediated resensitization impaired. MOR loses its responsiveness to agonists and gives rise to tolerance [1-3]. Since MOR phosphorylation plays such important roles in the development of sensitization and tolerance, attempts have been made to study the effects of serine and threonine phosphorylation in the C-terminus of MORs on receptor signaling using phosphorylation deficient S375A and T394A mutant MORs. In contrast to wild-type MORs, phosphorylation of T394A mutant human MORs (hMORs) expressed in Chinese hamster ovary (CHO) cells induced by the full opioid agonist, [D-Ala^2^-MePhe^4^-Gly-ol] enkephalin (DAMGO), is much reduced, and DAMGO-induced inhibition of adenylyl cyclase activity is also decreased [4,5]. Furthermore, the T394A rat MORs expressed in neuroblastoma cells showed faster internalization and rapid resensitization [6]. Similarly, morphine-induced desensitization was blocked in S375A-expressed HEK cells [7]. Thus, phosphorylation of T394 and S375 in MORs is critical in MOR sensitization, internalization and resensitization. Most of our understanding of desensitization and tolerance development are based on the studies of opioid actions in model cells or in tissues isolated from chronic opioid treated animals. Behavioral consequences of the mutation have not been studied extensively. Recently, S375A MOR knock-in mice were produced [8]. Behavioral tolerance was assessed in the mutant mice. Morphine-induced tolerance was found not to be affected by S375A mutation. Although elegant, the approach is labor intensive and time consuming. To find a simpler way to facilitate the determination of behavioral consequences of specific phosphorylation sites in MORs that may play a major role in the pain signal transmission, we intrathecally applied T394A MOR plasmid DNA to express mutant hMORs in sensory DRG neurons, which participate in the transmission of nociceptive information. The nociceptive behavioral consequences of mutant hMOR expression were then examined. This study allowed us to determine if such an approach can efficiently screen the properties of mutant receptors and assess the role of mutation sites in the function of receptors.

## Results

### Expression of hMOR-T and hMOR in DRG neurons through direct plasmid injection

To express mutant hMORs in DRGs, the hMOR-T (100 μg/25 μl) plasmid was slowly injected intrathecally through a syringe at the L4-5 vertebra level using the modified direct transcutaneous intrathecal method [9]. For positive control, hMOR plasmid was injected instead. For negative control, a GFP plasmid or saline vehicle was injected. No signs of adverse effects of the injection in the test animals were observed.

**Figure 1:**
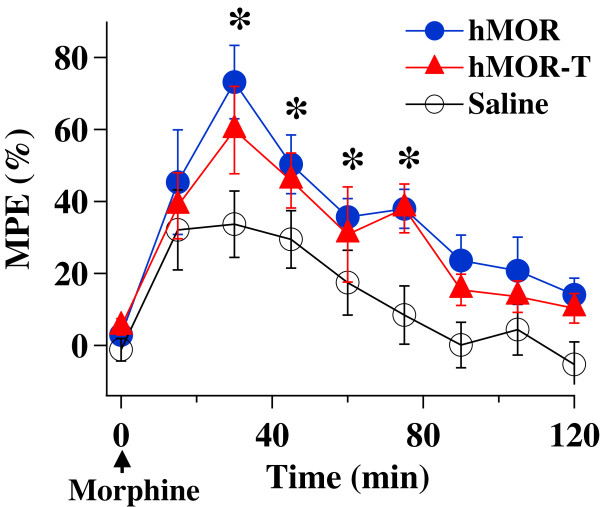
**Time course of morphine-induced analgesia.** A dose of morphine (4 μg/10 μl) was given at time 0 (arrow) and PWLs expressed in (% MPE) in response to heat stimuli were measured at various intervals. The analgesia was observed within 15 min of morphine application and reached its maximum level at 30 min. The analgesia was gradually decreased and dissipated >100 min later. The MPEs obtained from hMOR-T and hMOR rats were significantly higher than that of saline rats (*p < 0.05, n = 5-7). The differences in MPEs between hMOR-T and hMOR rats were not significant.

We first determined the functional consequence of the injection of hMOR-T or hMOR plasmid by studying the effects of morphine on thermal nociceptive responses. The development of analgesic effect of morphine was examined (Figure 1). Following an intrathecal injection of one dose of morphine (4 μg/10 μl), the paw withdrawal latency (PWL) of the rat hindpaw in response to heat stimulation was measured every 15 min for 120 min. The analgesic effect was observable 15 min after the injection, reached a peak level ~30 min and started to decay at 45 min after the morphine application. The PWLs gradually returned to the baseline level 90–120 min later. Morphine analgesia developed in a similar manner in all three rat groups except that the peak analgesia was significantly higher in hMOR-T and hMOR rats than in saline rats. In the subsequent experiments, unless specified, morphine effects were studied 30 min after its injection.

To confirm hMOR-T and hMOR expression in DRGs, rats that had undergone the behavioral studies were fixed and DRGs extracted. The level of exogenously expressed hMOR-T or hMOR in L4-5 DRGs was studied immunohistochemically using a monoclonal mouse anti-HA antibody, which recognized the HA epitope fused to the N terminus of hMOR-T and hMOR.

**Figure 2:**
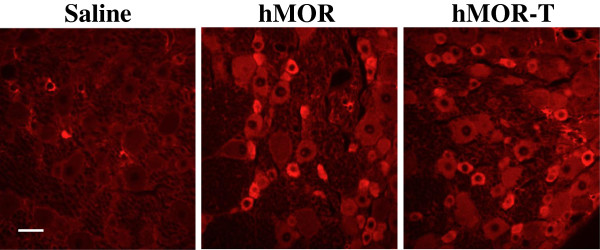
**Expression of HA in DRG neurons following i.t. injection of hMOR-T or hMOR plasmids.** HA immunostaining in DRG sections isolated from rats injected with saline, hMOR or hMOR-T plasmids. Bright HA immunofluorescence was observed in DRG neurons of both hMOR and hMOR-T injected rat groups. No HA labels were found in neurons of saline injected rats. Scale bars, 50 μm.

No HA labeled neurons were detected in the saline rat group. In contrast, 30-31% of DRG neurons were brightly labeled in hMOR-T and hMOR rat groups (Figure 2). Ten to eleven percent of the labeled cells were small diameter (d) (d < 25 μm) and 18-19% of them were medium size (25 ≤ d < 35 μm) DRG neurons (Table 1). Very few large neurons were labeled. The level of HA expression and the distribution of the expression among different size neurons were almost identical in the hMOR-T and hMOR rat groups. Since HA labels were not found in satellite glial cells, the neuron-specific endolase (NSE) or synapsin promoter incorporated in our plasmids effectively limited the hMOR or hMOR-T expression to neurons. Endogenous MORs, labeled with anti-MOR antibody, were found in 29 ± 4.5% of DRG small and medium size neurons (data not shown). Thus, plasmid injection substantially increased the level of MORs expressed in DRGs.

**Table 1:** HA-labeled hMOR and hMOR-T expressed in different sized DRG neurons

	**Small cell**	**Medium cell**	**Large cell**	**Total†**
	d* < 25 μm	***25 < d < 35 μm***	***d > 35 μm***	
Saline	0	0	0	0
hMOR	9.71 ± 0.66	18.22 ± 1.83	1.82 ± 0.24	29.75
hMOR-T	11.08 ± 0.17	19.14 ± 1.86	0.45 ± 0.45	30.67

Total number of cells counted as 100%; all values are HA-labeled cells expressed as % of counted cells. Values are means ± SE; 3 rats in each rat group.

*d: cell diameter. †Total: total percentage, including small, medium and large cells, of HA-labeled cells.

There were no significant differences between hMOR and hMOR groups in all sizes of DRG neurons.

### Morphine analgesia is enhanced in hMOR and hMOR-T rats

**Figure 3:**
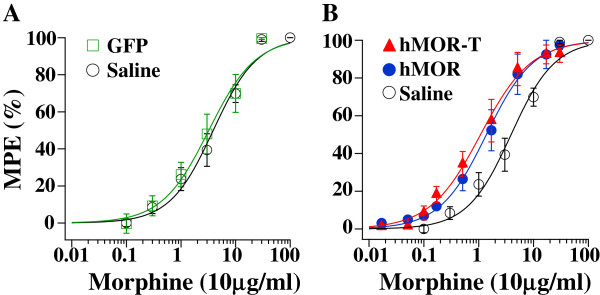
**Dose–response of morphine on PWLs in hMOR-T, hMOR and saline or GFP rats.** Different doses of morphine were given in 10 μl volume and the PWL responses were determined 30 min after each morphine application. The solid lines were the theoretical fit of the data using the Hill equation. **A**. The Hill coefficient (h) was 1.01 for GFP and 1.09 for saline rats. Morphine ED50 (μg/10 μl) was 3.23 ± 0.49 for GFP and 3.86 ± 0.53 for saline rats. **(B)** The h was 0.94 for hMOR-T, 1.02 for hMOR. The morphine ED50 was 1.08 ± 0.08 for hMOR-T, 1.38 ± 0.07 for hMOR. The apparent morphine affinities of hMOR-T and hMOR rats were significantly higher than that of saline or GFP rats.

The dose-responses of morphine in the four rat groups (saline, GFP, hMOR-T, hMOR, 5–7 rats for each group) were determined by examining the PWLs in the presence of different doses of i.t. morphine. One or two concentrations of morphine were studied in one experimental session. When two doses of morphine were tested, a cumulative morphine dose–response protocol with a starting dose and a fixed interval [10] was used. For instance, an initial dose of i.t. morphine, e.g., 0.1 μg/10 μl, was applied. Thirty minutes later, the PWL was measured and a second dose of i.t. morphine, e.g., 1 μg/10 μl was administered and measured after a 30 min wait. Rats were then returned to their cages to recover. Three days later, a second set of i.t morphine dosages, e.g., 0.3 and 3 μg/10 μl, were studied. Since the two morphine doses used in one experiment differed by 10 fold, the cumulative effect between two morphine applications was not significant. The dose–response curves obtained could well be fitted by the Hill equation and the effective doses (ED50) were obtained (Figure 3).

To make sure that plasmid itself did not produce non-specific effects on morphine responses, we compared the morphine dose–response in rats injected with GFP plasmid with those injected with saline. The dose–response curves obtained from GFP and saline rat groups were almost identical. The ED50 of morphine was 3.23 μg/10 μl for GFP rats and 3.86 μg/10 μl for saline rats (Figure 3A). Thus, the changes in morphine dose-responses produced by MOR or MOR-T plasmid were not a result of unspecific effects of plasmid injection. The ED50 of morphine for hMOR-T (1.08 μg/10 μl) and ED50 for hMOR (1.38 μg/10 μl) rats were similar (Figure 3B). Both were significantly lower than the ED50 for saline or GFP rats. Morphine was therefore much more potent in producing analgesia in plasmid-injected rats.

### Morphine-induced tolerance

**Figure 4:**
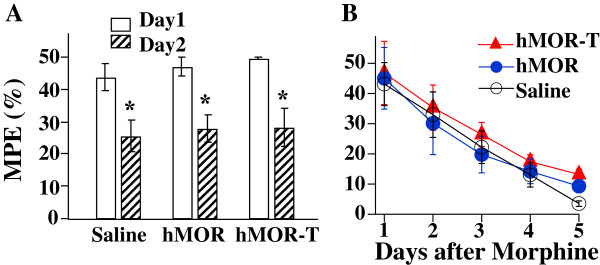
**Acute and chronic morphine behavioral tolerance in hMOR-T and hMOR rats are the same. A**. Acute tolerance was assessed by measuring changes in morphine analgesia induced by a high dose (30 μg/10 μl) of morphine given 18 hrs earlier. On day 2, the three rat groups exhibited a similar extent of reduction in morphine responses (saline: 41.4%, n = 4; hMOR: 40.4%, n = 3; hMOR-T: 43.3%, n = 3). **B**. Chronic morphine tolerance was studied in rats subjected to morphine treatment (hMOR-T: 1.5 μg/10 μl, n = 8; hMOR: 1.5 μg/10 μl, n = 4; saline: 5.0 μg/10 μl, n = 4) twice daily for 5 consecutive days. The chronic morphine-induced tolerance developed quickly and the rates of development among the rat groups were similar.

It has been shown that hMOR-T abolishes MOR phosphorylation, thus the DAMGO-induced desensitization of MOR [4,5]. It was therefore of interest to determine if this cellular change in MOR function results in any changes in morphine-induced behavioral tolerance. We first determined the acute tolerance by applying a high dose (30 μg/10 μl) of i.t. morphine and measuring PWLs on thermal stimuli. Eighteen hours later, the response to a second dose (30 μg/10 μl) of morphine was again measured. The morphine response was significantly reduced (Figure 4A). The extent of decrease was similar among hMOR-T, hMOR and saline rat groups. Thus, T394A mutation did not affect morphine-induced acute tolerance.

We then examined morphine-induced chronic tolerance in the three rat groups. Intrathecal morphine (1.5 μg/10 μl for hMOR-T and hMOR rats and 5.0 μg/μl for saline rats) was applied twice daily. The specific morphine doses were chosen to produce ~50% of the maximum possible effect (MPE) in respective rat groups. The first dose of morphine was given in the morning and the analgesic effect was subsequently measured. The second dose of morphine was given in the afternoon and rats were returned to cages. The dosing regiment was repeated for 5 consecutive days. The morphine analgesic effects were diminished rapidly with repeated morphine applications. The rates of decrease were similar among three rat groups. Thus, the upregulation of MORs and mutation of T394A altered neither the development nor the extent of morphine-induced chronic tolerance (Figure 4B).

### Differences and similarities between DAMGO- and morphine-induced analgesia

**Figure 5:**
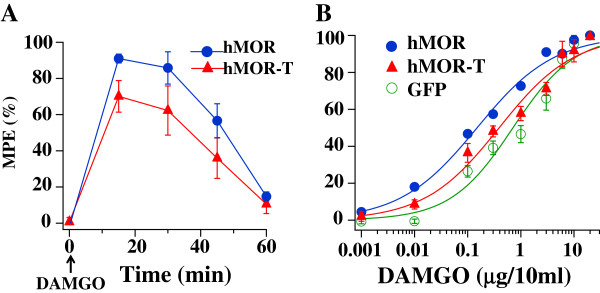
**Time course and dose response of DAMGO in hMOR-T and hMOR rats. (A)** DAMGO (3 μg/10 μl) was given at time 0 (arrow) and PWLs were measured at various intervals. The DAMGO induced analgesia developed quickly, reached a maximum ~15 min and the response returned to the baseline ~ 60 min later. **(B)** The DAMGO ED50 (μg/10 μl) obtained from dose–response analyses were 0.15 ±0.02 ((hMOR), 0.38 ± 0.08 (hMOR-T) and 0.66 ± 0.13 (GFP). The Hill coefficients were 0.6 for hMOR, 0.6 for hMOR-T and 0.7 for GFP.

Since the properties of opioid-induced analgesia often depends on the agonist used, the dose–response and tolerance of the full-agonist, DAMGO, in hMOR and hMOR-T rats were also studied. The development of analgesic effect of DAMGO is shown in Figure 5A. Compared with morphine, DAMGO was faster acting. The analgesic effect of DAMGO reached a peak level ~15 min after application and dissipated 60 min later. In the subsequent experiments, DAMGO effects were measured 15 min after its injection.

The dose–response curves for DAMGO were fit with the Hill equation (Figure 5B). The ED50 of DAMGO was 0.15 μg/10 μl for hMOR rats, 0.38 μg/10 μl for hMOR-T rats and 0.66 μg/10 μl for GFP rats. The ED50 for hMOR-T was 2.53 fold larger than that for hMOR. This is in contrast with morphine ED50, which had no statistic difference between hMOR-T and hMOR rat groups (Figure 3).

**Figure 6:**
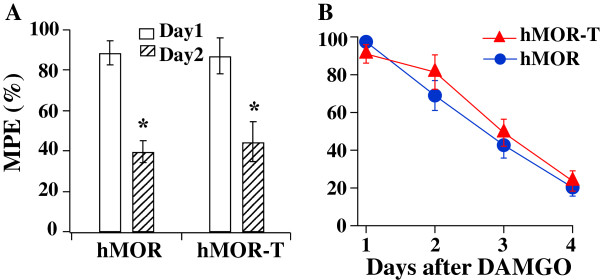
**hMOR-T and hMOR exhibit similar acute and chronic DAMGO-induced behavioral tolerance. (A)** Acute tolerance. On day 1, 4 μg DAMGO produced 88.6% MPE in hMOR rats and, 8 μg DAMGO produced 87.1% MPE in hMOR-T rats. On day 2, similar reduction in DAMGO responses (hMOR: 39.4%, hMOR-T: 44.4%, n = 5-7) were observed. **(B)** Chronic tolerance was obtained by applying 4 μg/10 μl DAMGO to hMOR and 8 μl/10 μl to hMOR-T twice daily for 4 consecutive days. The rates and extents of DAMGO-induced tolerance developed similarly in hMOR and hMOR-T rat groups.

Acute and chronic DAMGO-induced behavioral tolerance were also examined (Figure 6). We applied 4 μg/10 μl i.t. DAMGO to hMOR and 8 μg/10 μl to hMOR-T rats, which produced 88.6% and 87.1% MPE respectively. The same strength of second dose DAMGO was applied to each rat group 18 hrs later and behavioral responses were examined. Similar extent reduction of DAMGO responses in two rat groups was observed (Figure 6A). To examine DAMGO-induced chronic tolerance, 4 μg/10 μl or 8 μg/10 μl of DAMGO was applied to the hMOR or to hMOR-T rat group respectively twice daily and analgesic effects were measured after the 1st dose agonist application. The rate of development and the extent of tolerances between hMOR and hMOR-T groups in response to DAMGO application were very similar (Figure 6B). Thus, T394A mutation had no effect on DAMGO-mediated acute and chronic tolerance.

**Figure 7:**
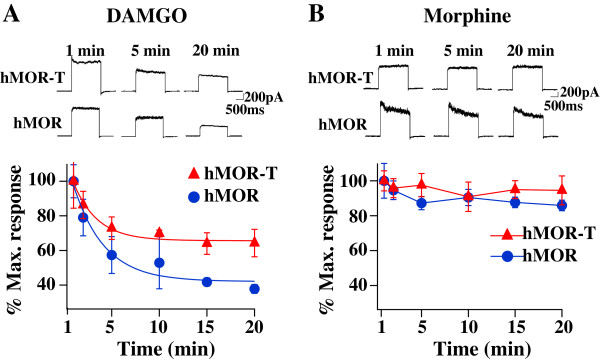
**T394 mutation is important in DAMGO-induced desensitization but does not alter morphine-induced I**_**K**_**. (A)** In the continued presence of DAMGO (10 μM), the amplitude of DAMGO-induced I_K_ decreased. The extent of desensitization in hMOR-T was less than that in hMOR. **(B)** In presence of morphine (30 μM), I_K_ did not inactivate.

To determine if the T394 site is important for MOR desensitization in DRG neurons, the amplitudes of agonist-induced outward I_K_ were measured during continued presence of DAMGO or morphine (Figure 7). DAMGO induced robust desensitization of I_K_ in both hMOR and hMOR-T expressed DRG neurons. Compared with hMOR-expressed DRG neurons, the extent of DAMGO-induced I_K_ desensitization in hMOR-T neurons was reduced. Thus, phosphorylation of the T394 site is important for full agonist-induced I_K_ desensitization in DRGs. Morphine-induced I_K_ desensitization was also studied. In contrast to DAMGO, morphine induced minimum I_K_ desensitization in DRG neurons isolated from hMOR and hMOR-T rats (Figure 7). Phosphorylation of the T394 site does not appear to affect morphine-induced I_K_ desensitization.

## Discussion

The purposes of this study are to determine (1) if the T394 site in MORs is involved in opioid tolerance and (2) if the application of DNA plasmids to upregulate receptors is a feasible way to study behavioral consequences of the expression of mutant receptors in sensory neurons. Viral vectors and mutant mice are two approaches commonly used to alter receptor expression. Aside from the concerns of the toxicity of viral vectors and possible compensatory adaptations in mutant mice, those approaches are complex. We sought a simpler method to accomplish the goal. Using hMOR as an example, we introduced mutant hMOR-T and hMOR into L4-L5 DRGs by intrathecal injection of DNA plasmid. A neuron-specific promoter was used to limit exogenous hMOR expression in neurons (Figure 2). Receptor upregulation could be seen within 3 days following the plasmid injection. The expression of hMOR-T and hMOR were found in small and medium DRG neurons which are known to mediate nociception. The time course of morphine effect was not altered by the mutation (Figure 1). Compared with GFP-injected rats, the upregulation resulted in 3.0- and 2.3-fold increase in morphine potency in hMOR-T and hMOR rat groups respectively (Figure 3), a magnitude analogous to that observed in DRGs transduced with adeno-associated viral vector (AAV) expressing MORs [11]. We showed here that the development of acute and chronic morphine behavioral tolerance is independent of the phosphorylation of T394 in MOR (Figure 4), a result similar to the findings obtained in S375A MOR mutant mice [8]. Thus, phosphorylation of the single site, i.e., T394 in hMORs, is not sufficient to alter the tolerance effect of morphine *in vivo.*

Even though our plasmid injection resulted in a substantial increase in the expression of hMORs in DRG neurons (Figure 2), one needs to ascertain that similar reduction in the morphine ED50 and identical morphine-induced tolerance observed in hMOR and hMOR-T DRG neurons were not a result of endogenous rat MORs masking morphine effects in the T394 mutant. The studies were therefore repeated using the full agonist, DAMGO. We found that DAMGO ED50 (Figure 5) and DAMGO-evoked I_K_ desensitization (Figure 7) in hMOR and hMOR-T DRGs are significantly different. Thus, T394 is important in determining DAMGO affinity and desensitization. The observations also suggest that the expression of hMOR-T in DRGs was sufficiently large to exhibit its opioid agonist-dependent responses. Since the development and extent of DAMGO-induced tolerance were similar in hMOR and hMOR-T neurons (Figure 6), as observed in morphine-induced tolerance (Figure 4), T394 in the hMOR appears to play a limited role in opioid-dependent tolerance.

Birdsong et al. [12] reported that prolonged treatment of cells with high-efficacy agonists, which causes MOR desensitization, increases the agonist affinity for MOR. In our study of opioid dose–response relation in hMOR and hMOR-T, DRGs were exposed to agonists longer than 15–30 min. Thus, the dose–response curves were obtained under desensitization conditions. We found that DAMGO had a lower affinity for hMOR-T than that for hMOR (Figure 5), a result consistent with the observation that DAMGO induced less desensitization in hMOR-T cells (Figure 7).

The advantage of our approach includes that DNA plasmids are easy to produce. With proper promoter design, the mutant receptors expression can be targeted to specific cell types (Figure 2). In contrast to the long waiting period (> 3 weeks) for viral vector-induced expression of mutant MORs, the expression reaches its peak level within 3 days of plasmid injection. Using a slow rate of intrathecal plasmid injection procedure (see Methods), the expression of mutant MORs can be largely confined within L4-6 DRGs. It has been well documented that DNA plasmids are seldom incorporated into the genome, have low immunogenicity and rarely produce ill effects in host animals [13]. There are a couple of noticeable limitations to the plasmid injection approach. One is that the expression level of mutant receptors is maintained for a short period. Following a single injection of i.t. hMOR-T or hMOR, the enhancing effect was reduced by 40% in ten days and dissipated in fourteen days. It has been shown that two injections of anti-inflammatory cytokine IL-10 DNA would prime immune responses and result in long term (up to 90 days) upregulation of IL-10 [13,14]. Unlike the immune mediators, double injections of mutant MOR DNA did not lengthen the duration of the expression (unpublished observation). Nevertheless, the expression of exogenous receptors could be maintained at a steady level following repeated injections of the DNA plasmid every seven days (data not shown). Another possible limitation is that changes induced by the mutant MOR have to be sufficiently large to overcome the influence of endogenous MORs. Since the differences in DAMGO ED50 and I_K_ desensitization in hMOR-T and hMOR DRGs were readily observed (Figures 5 and 7), changes in opioid responses induced by mutant hMORs are likely to be detected under our experimental conditions.

We and others have found that the mutation of a single phosphorylation site, i.e., T394 or S375, was not sufficient to alter chronic behavioral tolerance (Figures 4 and 6) [8]. It is of interest to determine if phosphorylation of other or multiple sites in MOR would alter morphine tolerance. In addition to T394 and S375, a number of serine and threonine sites, e.g., T357, S363 and T370, have been localized in the C-terminus of MOR [15-17]. In model cells, these sites were found to be phosphorylated by different kinases. In a mass spectrometric analysis phosphorylation of the T394 site was not detected [15]. This result is inconsistent with the observation that DAMGO-induced phosphorylation of T394A mutant receptors is much reduced [4,18]. Additional studies are needed to resolve the inconsistency. The consequences of the phosphorylation of serine and threonine sites in receptor desensitization have been extensively studied *in vitro*. The opioid-induced desensitization was found to diminish in T394A and S375A mutant receptors [4,6,7,18]. Furthermore, the mechanism underlying receptor desensitization depends on the agonist used [19-21]. In locus ceruleus neurons, morphine-induced desensitization was shown to depend on PKC whereas DAMGO-induced desensitization is mediated by GRK2 and independent of PKC [20]. It is suggested that DAMGO- and morphine-bound MORs assume differential conformations that are targeted by different GRKs to give rise to various efficiencies in receptor internalization [16,21,22] and changes in the responses to PKC [23]. We also found that I_K_ desensitization in hMOR expressing DRG neurons depends on opioid agonists. T394 mediates DAMGO-induced desensitization, but does not affect morphine action on I_K_ (Figure 7). The mechanism underlying the agonist-dependent desensitization and its effect on opioid-induced analgesic behaviors have yet to be explored.

The relationship between MOR desensitization and cellular tolerance has been studied in cells or slices obtained from chronic morphine treated animals [2]. Cellular tolerance was defined as a sustained decrease in the morphine efficacy for its coupling effector, e.g., GIRK channels [24] or as right-shift in the morphine dose–response curve [25]. Cellular tolerance and desensitization were found to be separate processes that occur simultaneously [24] and β-arrestin2 was shown to impair the MOR resensitization and recycling [25,26]. The relationship between cellular and behavioral tolerance is not entirely clear. The information will be essential for our understanding of the mechanisms underlying behavioral tolerance. Using our approach, the behavioral tolerance of various mutants can be determined and its relationship with cellular tolerance and desensitization assessed. This knowledge will be useful in designing ways to improve morphine usage in clinics.

## Conclusions

Using a plasmid to deliver mutant T394A hMOR to DRG neurons, we showed that the T394 site mediates DAMGO-induced I_K_ desensitization and alters DAMGO potency for hMOR while it does not affect morphine-induced responses. Furthermore, phosphorylation of the T394 site had a limited role in the development of DAMGO- and morphine-induced tolerance. More important, the study demonstrates that nociceptive behavioral consequence of mutations in receptors can be efficiently studied by plasmid gene delivery of mutant receptors to sensory neurons.

## Methods

### Animals

Experiments were performed on adult (200–250 g) male Spargue Dawley rats. All experimental procedures were approved by the Institutional Animal Care and Use Committee at the University of Texas Medical Branch and were in accordance with the guidelines of the National Institutes of Health and of the International Association for the Study of Pain (IASP).

### Plasmid preparation

The N-terminus HA epitope (YPYDVPDYA) tagged wild-type human mu opioid receptor (hMOR) plasmid and the point mutant (T394A) of the HA tagged hMOR plasmid (hMOR-T) used in this study were gifts from Dr. J.B. Wang, University of Maryland, Baltimore, MD. To produce plasmids containing either of the opioid receptor genes under the control of an neuron-specific endolase (NSE) or synapsin promoter, the PCR products of the hMOR-T and of hMOR were prepared and ligated into the pTR-NSE or -synapsin plasmid as described [27].

To upregulate MORs in rat DRGs, the hMOR-T (100 μg) plasmid was suspended in a 25 μl sterile sucrose (3%)-containing Dulbecco’s phosphate-buffered saline [14] and injected intrathecally through a syringe at the L4-L5 vertebra level using the modified direct transcutaneous intrathecal method [9]. The plasmid solution was slowly injected within 5 seconds and the needle stayed for another 5 seconds before removal. In some experiments, a second dose of intrathecal plasmid was administered two days later. For controls, hMOR, GFP or saline vehicle was injected.

### Behavioral experiments

The opioid-agonist induced analgesic effects on thermal hyperalgesia to radiant heat in different rat groups were assessed as described [27]. To obtain consistent behavioral responses, rats were acclimated in Plexiglass boxes placed on the test platform for 30 minutes per day for 3 days prior to experiments. For morphine or DAMGO applications, a PE-10 catheter was placed between the vertebrae S1 and S2 with its tip extended rostrally to the vertebrae L4-L5 under sodium pentobarbital anesthesia. To measure PWLs, radiant heat was placed under the plantar surface of the hind paw and the time elapsed from the onset of radiant heat stimulation to the withdrawal of the hind paw was recorded. The heat intensity was adjusted to give a baseline latency around 10s; a cutoff time of 30s was set to prevent possible tissue damage. To obtain baseline PWLs, three measurements separated by a 5-min interval were made for the rat’s hindpaw and scores were averaged.

Two days after the last plasmid injection, a PWL was determined prior to morphine or DAMGO administration. To determine the time course of opioid agonist effects, a dose of i.t. agonist was given and the withdrawal latencies were measured at various time points later. To determine the dose-dependent effect of an agonist on PWLs, either one or two doses of the agonist was used. When a cumulative agonist dose–response protocol [10] was used, an initial dose of i.t. agonist was given. The withdrawal threshold was measured 15 (for DAMGO) or 30 min (for morphine) later. A second dose of agonist was then applied and PWLs were measured.

### Immunohistochemistry

Rats were perfused with 4% paraformaldehyde and 0.2% picric acid fixative within 2 days after the end of the behavioral study. L4, L5 DRGs were removed, sectioned (10 μm) and washed with phosphate buffer saline (PBS). The sections were incubated with a PBS solution containing 10% normal goat serum and 0.2% Triton X-100 for 60 minutes to block non-specific binding. To detect exogenously expressed hMOR-T or hMOR receptors, sections were incubated with a monoclonal mouse anti-HA antibody (1:1000, Sigma, St. Louis, MO) diluted in a 0.2% TritonX-100 PBS plus 4% normal goat serum at 4°C overnight. In control staining, the primary antibody was omitted. The primary antibody was removed by rinsing the tissue sections in the PBS solution for 5 minutes × 3 times. The sections were then incubated with the secondary antibody, Alexa Fluor 546 goat anti-mouse IgG (1:200, Molecular Probes, Carlsbad, CA) in 0.2% Triton-X PBS plus 4% normal goat serum, at room temperature for 1 hour. The sections were washed 5 minutes × 3 times and mounted with mount medium (Vector Laboratories, Burlingame, CA). Labeled cells from fifteen tissue sections obtained from each rat were counted and results from 3 rats in each rat group, i.e., hMOR-T, hMOR, saline rats, were analyzed. MetaMorph offline software was used for digital image data analysis.

### Opioid tolerance

Morphine- and DAMGO-induced tolerances were studied by determining PWLs following repeated applications of morphine. Baseline PWLs were first determined prior to the application of an opioid agonist. Acute tolerance was determined by measuring changes in analgesia following two high dose of agonist treatments applied within 18 hours. For chronic tolerance studies, opioid agonist was i.t. injected twice a day (once in the morning and once in the afternoon). Thermal-induced PWLs were assessed following the morning morphine dosing. The procedure was repeated for 4–5 days.

### Whole cell patch clamp recordings

Outward I_K_ were recorded from small or medium (15–30 μm in diameter) acutely dissociated DRG neurons isolated from 25–35 old male rats using the described method [28,29]. Briefly, L4-L5 DRGs were excised from anesthetized rats 3–5 days after plasmid injection and put in an ice-cold oxygenated dissecting solution. It contained (in mM) 135 NaCl, 5 KCl, 2 KH_2_PO_4_, 1.5 CaCl_2_, 6 MgCl_2_, 10 glucose and 10 HEPES, pH 7.2 (osmolarity, 305 mosmol/liter). After removal of the connective tissue surrounding the ganglia, the tissue was put into a dissecting solution containing papain (20 unit/ml, Worthington Biochemical, Lakewood, MJ) and collagenase D (1 mg/ml, Roche Applied Science, Indianapolis, IN) and incubated at 37°C for 50 min. After the incubation period, DRGs were removed from the enzyme solution, washed and placed into a fresh dissecting solution. Cells were then dissociated by trituration with fire-polished glass pipets and placed on glass coverslips. After a resting period (~ 2 h), a glass coverslip was put in the recording chamber and cells were superfused at room temperature with an external solution containing (in mM) 130 NaCl, 5 KCl, 2 KH_2_PO_4_, 2.5 CaCl_2_, 1 MgCl_2_, 10 HEPES, 10 glucose, pH 7.3; osmolarity, 295–300 mosmol/liter). During agonist application, the external solution containing a MOR agonist will be applied to the recorded cell through an electronic valve. Patch-clamp electrodes were filled with an internal solution, containing (in mM) 115 K-gluconate, 10 NaCl, 10 EGTA, 2 CaCl_2_, 1.5 Na-ATP, 0.3 GTP, 10 glucose and 10 HEPES and had a resistance of 3–5 Mohm. The currents were filtered at 5 kHz and sampled at 1 msec/pt using a Dagan amplifier. Current responses were analyzed using Igor Pro software.

Membrane potential was held at −60 mV. I_K_ responses were evoked by stepping from −60 mV to 0 mV and outward currents were recorded. Desensitization of I_K_ was quantified by measuring changes in peak outward K^+^ currents during a prolonged application of a MOR agonist. The change in I_K_ was expressed as the percentage change of the initial peak response.

### Data analyses

All data were recorded as mean ± SEM. Analgesic effects were shown as MPE by using the relation MPE = (postdrug PWL - baseline PWL) / (cutoff PWL - baseline PWL) × 100%. To assess the significance of changes between two means, the Student’s *t* test was used. The time course and chronic tolerance of morphine effects were analyzed by two-way ANOVA with repeated measures followed by post-hoc Bonferroni analysis. Dose–response curve was plotted as MPE vs morphine dose and fitted with the Hill equation, i.e., *response* = min + (max − min) * [*dose*^*n*^(*dose*^*n*^ + *ED*50^*n*^)] where min and max are minimal and maximal responses, the potency, ED50, is the effective morphine dose producing 50% of the maximal analgesic effect, and *n* is the Hill coefficient. A *P <* 0.05 was considered significant.

## Abbreviations

CHO: Chinese hamster ovary cells; DAMGO: [D-Ala^2^-MePhe^4^-Gly-ol] enkephalin; DRG: Dorsal root ganglion; hMOR: Wild-type human mu-opioid receptor; hMOR-T: T394A mutant human mu-opioid receptor; MOR: Mu-opioid receptor; MPE: Maximum possible effect; PWL: Paw withdrawal latency.

## Competing interests

The authors declare that they have no competing interests.

## Authors’ contributions

GL designed and performed behavioral and electrophysiological experiments, analyzed the data and edited the manuscript, FM designed and performed immunocytochemical experiments, analyzed the data and prepared the manuscript. YG analyzed the data and edited the manuscript. LMH conceived and coordinated the study, designed experiments, prepared plasmids, analyzed the data and prepared the manuscript. All authors read and approved the final manuscript.
